# Information Use Differences in Hot and Cold Risk Processing: When Does Information About Probability Count in the Columbia Card Task?

**DOI:** 10.3389/fpsyg.2015.01727

**Published:** 2015-11-18

**Authors:** Łukasz Markiewicz, Elżbieta Kubińska

**Affiliations:** ^1^Center of Economic Psychology and Decision Sciences, Economic Psychology, Kozminski UniversityWarsaw, Poland; ^2^Department of Financial Markets, Cracow University of EconomicsKraków, Poland

**Keywords:** Columbia Card Task (CCT), Cognitive Reflection Test (CRT), dual process theory, dynamic risk taking, experience based probability format, information use

## Abstract

**Objective:** This paper aims to provide insight into information processing differences between hot and cold risk taking decision tasks within a single domain. Decision theory defines risky situations using at least three parameters: outcome one (often a gain) with its probability and outcome two (often a loss) with a complementary probability. Although a rational agent should consider all of the parameters, s/he could potentially narrow their focus to only some of them, particularly when explicit Type 2 processes do not have the resources to override implicit Type 1 processes. Here we investigate differences in risky situation parameters' influence on hot and cold decisions. Although previous studies show lower information use in hot than in cold processes, they do not provide decision weight changes and therefore do not explain whether this difference results from worse concentration on each parameter of a risky situation (probability, gain amount, and loss amount) or from ignoring some parameters.

**Methods:** Two studies were conducted, with participants performing the Columbia Card Task (CCT) in either its Cold or Hot version. In the first study, participants also performed the Cognitive Reflection Test (CRT) to monitor their ability to override Type 1 processing cues (implicit processes) with Type 2 explicit processes. Because hypothesis testing required comparison of the relative importance of risky situation decision weights (gain, loss, probability), we developed a novel way of measuring information use in the CCT by employing a conjoint analysis methodology.

**Results:** Across the two studies, results indicated that in the CCT Cold condition decision makers concentrate on each information type (gain, loss, probability), but in the CCT Hot condition they concentrate mostly on a single parameter: probability of gain/loss. We also show that an individual's CRT score correlates with information use propensity in cold but not hot tasks. Thus, the affective dimension of hot tasks inhibits correct information processing, probably because it is difficult to engage Type 2 processes in such circumstances. Individuals' Type 2 processing abilities (measured by the CRT) assist greater use of information in cold tasks but do not help in hot tasks.

## Introduction

Decision theory describes risky situations as choices between lotteries characterized by outputs (gains and/or losses) and their probabilities. A rational agent making decisions in compliance with expected utility theory (von Neumann and Morgenstern, 1953) should concentrate on each parameter of a decision equally, thus taking into consideration all available information. The assumption that outcomes (or functions of them) are weighted by their probability underlies most economic and behavioral theories. On the other hand, studies constantly show that cognition is difficult and costly, partially because of peoples' limited processing capacity, including attention (Chabris and Simons, 2011). Among other things, this is why decision makers first try to simplify a decision problem in the so called “editing phase” (Kahneman and Tversky, 1979). Because of attention limitations they do not use all of the available risk information when making a decision. Therefore, many research paradigms (e.g., Active Information Search, Englander and Tyszka, 1980; Huber et al., 1997 and Mouselab) investigate the order of information acquisition (Schulte-Mecklenbeck et al., 2011).

However, relatively little has been said about differences in information use in affective (hot) and cognitive (cold) risk processing. Do people concentrate on different risk characteristics (losses of, and gains on, stakes, and their probabilities) in emotional risk taking (e.g., parachute jumping) compared to cognitive risk taking (e.g., pension scheme decisions)? Some studies (Pachur et al., 2014; Suter et al., 2015) have demonstrated that the impact of probabilities is strongly diminished for affect-rich outcomes. However, these studies used outcomes of different valences, assuming that medical outcomes are affect-rich and that monetary outcomes are affect-poor. Thus, they could have detected a domain difference (Sawicki and Markiewicz, 2015) rather than an affect magnitude difference. The assumption that probabilities have a diminished impact in affect-rich outcomes has solid foundations: Rottenstreich and Hsee (2001) and Sunstein (2002) both noted that affect-related decisions are insensitive to probabilities, however, here again, affect differences were mostly combined with different domains, this confound possibly leading to an error in the conclusions drawn. Thus, our present research question asked whether there are differences in the importance of risky situations' parameters (gain/loss/probability) in hot and cold decision processes within a single domain.

To explore differences in affective (hot) and cognitive (cold) risk processing within a single domain, Hot, and Cold versions of the Columbia Card Task (CCT) are used (Figner and Voelki, 2004; Figner et al., 2009). In each of the 63 rounds of both versions of the computerized CCT a participant (P) is informed about the number of loss cards (1, 2, or 3) hidden among 32 cards, the point value associated with each loss card (250, 500, or 750), and the point value associated with gain cards (10, 20, or 30)^1^. In the Cold version of the CCT, participants state in advance how many cards they want to turn over, in the Hot version participants turn over cards one-by-one until they decide to finish. In the Hot version, participants receive feedback immediately after turning over a card, while in the Cold version they receive feedback at the end of the final (63rd) round. P's task is to turn over cards and to achieve as great a total gain as possible at the end of the final round. Only 9 of the 63 rounds are generated randomly. The remaining 54 are programmed in such a way that loss cards appear at the end of the round (participants can choose cards safely for up to 32 cards minus the number of loss cards). The average number of turned over cards in the 54 rigged feedback rounds is taken as a measure of risk taking.

Apart from assessing risk taking, the CCT allows the calculation of “information use” Figner et al. (2009): the impact of three risk parameters (the gain amount, loss amount, and the probability of gains) on the risk taking behavior of an individual. To date, CCT studies have concentrated mostly on risk taking propensity (the number of turned over cards) in adults and adolescents, risk taking propensity's neural correlates (van Duijvenvoorde et al., 2015), risk taking propensity and emotion regulation strategies' correlates (Panno et al., 2012), the relationship of CCT risk taking propensity with personality traits (Penolazzi et al., 2012), and other risk measurement scores (Buelow and Blaine, 2015). In the CCT research paradigm papers usually report risk propensity scores, consideration of information processing is often omitted (Panno et al., 2012; Huang et al., 2013). However, CCT studies which have calculated information use demonstrate that it is generally greater in cold situations than in hot situations (Figner et al., 2009; Buelow, 2015). Here, though, we investigate which type of risk-related information matters most in cold and hot processes. Risky situations are traditionally defined by a set of characteristics involving payoffs and their probabilities, and loss levels, and their probabilities (von Neumann and Morgenstern, 1953). Thus, we were interested in whether higher information use in cold processes results from general or selective concentration on risky situation characteristics.

Pachur et al. (2014) claim that in affect-poor situations decision makers commonly rely on a compensatory process, trading off outcomes, and probabilities, whereas in affect-rich choices people more often rely on a non-compensatory heuristic process. Thus, in cold tasks decision makers should place significant weight on all three risk parameters (probability, gain amount, loss amount), while in hot tasks they should use a simplified approach and concentrate on one of the risk parameters. We therefore hypothesized that (H1) decision makers place different weights on risky situation parameters (gain amount, loss amount, and probability of gains), while making risky decisions in cold and hot tasks.

As defined by Figner et al. (2009), information use considers the number of significant parameters (using a dummy variable resulting from an ANOVA conducted for each participant for each parameter of a risky situation separately). This does not enable comparison of the decision weights for the decision parameters considered. Thus, to test H1 we developed a new way of measuring information use based on axiomatic Conjoint Measurement Theory (CMT; Luce and Tukey, 1964; Krantz et al., 1971) and discussed in detail in Section The Columbia Card Task (CCT). This new approach uses ordinary least squares (OLS) regression to explain the number of cards turned over in a given round in terms of the round's risk characteristics. Transformed regression betas serve as separate decision weights (summing to 1) for probability, loss, and gain amount—all expressed on an interval scale to provide an effective test of H1.

We also investigated factors that make people use risky situation-related information. In the view of dual-process theories, the mind operates using two types of processes: effortless and automatic processes (Type 1 processes, see Evans and Stanovich, 2013) and effortful, rule-based processes which use working memory resources (Type 2 processes). The most prominent model concerning the interplay between these systems is the “Default Interventionist” model, which suggests that Type 2 processes can intervene and override intuitive Type 1 responses when feelings as to the correctness of the intuitive answer are troublesome (Thompson et al., 2013)—see also alternative models such as those of Handley and Trippas (2015) and Pennycook et al. (2015). Further, the engagement of Type 2 explicit processes, which determine information use, depends on task characteristics (Rolison et al., 2011, 2012). We speculate that high emotional arousal associated with the CCT Hot version decreases the chance of Type 2 process usage compared to the CCT Cold version. If so, an individual's abilities to use Type 2 explicit processes (cognitive abilities such as numerical skill or working memory use) should predict information use only in the Cold, but not the Hot, condition. Likewise, it would be expected that WISC–III backward digit span test scores (which measure the storage and manipulation of meaningless numerical information: Wechsler, 1991) would be significantly positively correlated with information use in the Cold, but not the Hot, condition. However, previous research shows a positive correlation for both conditions (Figner et al., 2009), although the same study also demonstrated that tests of higher order executive functions (the Key Search Task, Zoo Map Test, Water Test (all from the Behavioral Assessment of Dysexecutive Syndrome task battery: BADS–C), which involve skills such as planning, novel problem solving, inflexibility, and perseveration, correlate with information use in the Cold, but not the Hot, condition. All these skills are assumed to be a function of involvement of the dorsolateral region of the prefrontal cortex (Cohen, 2005; Figner et al., 2009). Similarly, performance on the Wisconsin Card Sorting Task (WCST), which measures executive functions (such as abstract thinking, problem solving, perseveration, and cognitive set shifting), has been found to correlate with separate information use scores only in the Cold condition (Buelow, 2015). Because of these contradictory results we used another measure of cognitive ability: the Cognitive Reflection Test, CRT (Frederick, 2005), which uses three simple numeracy questions to assess an individual's ability to resist reporting the first response that comes to mind (generated by Type 1 processes) in favor of a reflective correct answer (generated by Type 2 processes). Thus, H2 was as follows: information use in the Cold condition is related to an individual's ability to resist Type 1 processing cues. However, no such relationship was expected in the Hot condition. In other words, the higher the ability to override Type 1 with Type 2 cues (as shown by CRT score), the higher is the ability to process all information (as indicated by the information use parameter in the CCT) in cold risky decision-making. We speculate that responding in the Hot task reflects a failure of Type 2 processing to become engaged (i.e., emotions block the possibility of overriding Type 1 with Type 2 cues): under such circumstances even reflective people should not use more information.

In the studies below we tested both hypotheses. Two studies are presented. Study 1 tests both H1 and H2, but Study 2 tests the robustness of H1 alone. H1 is tested by comparing decision weights for probability, loss amount, and gain amount across the hot and cold conditions, while H2 is tested by examining correlational patterns.

## Methods

### Study 1

Study 1 was conducted to verify both H1 and H2.

#### Measures

##### The columbia card task (CCT)

Hot and Cold versions of the CCT^2^ (Figner and Voelki, 2004; Figner et al., 2009) were used to track the deliberative (cold) and affective (hot) processes involved in risk taking, and thereby to test the two hypotheses.

In each of 63 computer-generated rounds, a participant sees a deck of 32 cards (4 rows, 8 cards per row) face down. The participant is informed about the particular characteristics of a round: the number of loss cards (*n*) hidden among all the remaining gain cards (32-n), the monetary amount associated with each loss card, and the amount associated with the gain cards. In both versions, the participant (P) faces a similar decision problem, namely how many cards to turn over out of 32. In the Hot task, the participant is provided with both win/loss feedback after each card is turned over and feedback on the number of points after each single round. In each round of the Hot task, the participant points to a face-down card to turn it over and reveal its face. If the card is a gain card (a smiling face), the gain is added to the total game balance, and then the participant points to the next card. The participant can turn over cards until they decide that the risk of turning over the next card is too high *or* until they encounter the loss cards. Contrary to the Hot version, the Cold task only provides points feedback when the participant completes the entire task. The participant does not point to particular cards in the Cold task, but needs to decide how many of the 32 cards to turn over in the particular round that is described by the number of loss cards, loss amount, and gain amount. Furthermore, the participant knows that a draw will be made by the computer after they complete the entire task of 63 rounds. Only these rigged feedback rounds are used as an indicator of risk preference (measured as the average number of turned over cards in all 54 rigged rounds) and information use, which is quantified as the number of statistically significant differences between groups defined by three categorical variables: probability of loss, loss amount, and gain amount. With the initial value of information use set to 0, a value of 1 is added to the information use parameter if an ANOVA calculated separately for each respondent and each type of risk information reveals a significant main effect (at a significance level of *p* < 0.05).

The previously proposed information use parameter (Figner et al., 2009) does not enable comparison of the decision weights of risky decision parameters (gain, loss, and probability) at the individual level. Thus, we employ a conjoint analysis methodology (Orme, 2010), rooted in CMT (Luce and Tukey, 1964; Krantz et al., 1971), to propose a new way of measuring information use.

By analyzing individual choices with OLS regression [rather than ANOVA as proposed by Figner et al. (2009)] we obtain *R*^2^coefficients specific to individual respondents, reflecting the extent of information's influence on risk taking. The OLS regression is calculated separately for each individual covering all 54 answers in rigged rounds, where the number of turned over cards serves as the dependent variable and the relevant round characteristics (gain level, loss level, and probability) are recoded into dummy variables—each defined by one characteristic within three possible levels of: gain (10, 20, 30), loss (250, 500, 750), and probability (1, 2, or 3 hidden loss cards)—which serve as independent variables. The independent variables are binary variables taking values of 0 or 1, thus, to avoid correlation of predictors (e.g., if the “2 hidden card” variable takes a value of 0, and the “3 hidden card” variable takes a value of 0, the “1 hidden card” variable must have a value of 1) each initial variable (“10 gain”; “250 loss,” and “1 hidden card”) is excluded from the regression equation following a common rule (Orme, 2010).

The regression *R*^2^ explains how well the choice parameters (gain level, loss level, and probability) explain the number of turned over cards. Thus, it can serve as an alternative information use parameter–and potentially as an additional parameter for screening non-rational respondents and those who did not understand the task. Based on the regression coefficients (betas) we derived part-worth utilities. At the very beginning, the coefficients for the first levels of each attribute were derived from the intercept, as one third of this coefficient. One third of the intercept was also added to betas associated with the two remaining levels of attributes. Finally the “zero-centered diffs,” standardization procedure (Sawtooth Software, 2012) was used to compare beta values between participants. The zero-centered diffs procedure is simply a rescaling transformation performed for each respondent. First, the mean of the betas for the three levels for every attribute is subtracted from each beta to obtain zero-centered data. Next, the coefficients are multiplied by a factor such that the sums of the three differences in the best and worst levels of each attribute is equal to the product of the number of attributes (*k*) and 100. This gives an average 100 point range in beta coefficients for every attribute. The final vector of beta coefficients is reported as part-worth utilities for every attribute level and these take values within the same interval scale.

Additionally, following a conjoint procedure, we calculated the relative importance score (RIS) for each attribute–this variable being measured on a ratio scale. The role of an attribute in making a decision is measured by the range of its levels of part-worth utilities. The RIS is the percentage that the range of part-worth utilities for a certain attribute constitutes in the sum of ranges of part-worth utilities for all attributes. RIS sum to 100% and determine the relative impact of each attribute in the final decision of each respondent, thus they are often called “decision weights.” Finally, we also calculated the following variables: Loss amount INDEX; Gain amount INDEX; Probability INDEX. Thus, each index was multiplied by the product of its RIS and its coefficient of determination (*R*^2^). This new way of measuring information makes it possible to ascertain which information type matters most in risky decision making—and allows comparisons between respondents based on this.

To illustrate this novel conjoint approach to CCT data analysis, let us consider responses given by an arbitrarily chosen participant in the rigged rounds shown in Table 1. Following Figner et al. (2009), three One-way ANOVAs should be run, where independent variables (each with three levels) are Loss cards, Gain amount, and Loss amount, and the dependent variable is the number of turned over cards. Information use in this approach is the number of ANOVAs that detect statistically significant differences among number of turned over cards for the three independent variables.

**Table 1:** **The example of an arbitrarily chosen participant's responses on the CCT**.

**Round No**.	**Turned over cards**	**Loss cards**	**Gain amount**	**Loss amount**
1	15	3	10	750
2	32	2	10	750
3	15	2	30	250
4	24	1	10	250
5	7	2	20	500
6	15	2	20	750
…				
53	12	1	10	500
54	26	2	20	750

Data from Table 1 is then recoded, as shown in Table 2, to run OLS regression. There are two dummy variables for Loss cards, namely Loss cards (2), Loss cards (3), level 1 being implicitly coded in these two dummy variables. If the Loss cards variable takes a value of 1, then both Loss cards (2) and Loss cards (3) are 0; if Loss cards is 2, then Loss cards (2) is 1, while Loss cards (3) is 0; and finally if Loss cards is 3, then Loss cards (2) is 0, while Loss cards (3) is 1. The variables Gain amount (20), Gain amount (30), Loss amount (500), and Loss amount (750) are created similarly.

**Table 2:** **Transformed data from Table 1 for OLS regression**.

**No round**	**No turned over cards**	**Loss cards (2)**	**Loss cards (3)**	**Gain amount (20)**	**Gain amount (30)**	**Loss amount (500)**	**Loss amount (750)**
1	15	0	1	0	0	0	1
2	32	1	0	0	0	0	1
3	15	1	0	0	1	0	0
4	24	0	0	0	0	0	0
5	7	1	0	1	0	1	0
6	15	1	0	1	0	0	1
…							
53	12	0	0	0	0	1	0
54	26	1	0	1	0	0	1

Let us assume that *R*^2^ = 0.367 and regression coefficients are as follows: β_0_ = 29.537; β_LossCard_2_ = −1.278; β_LossCard_3_ = −2.333; β_GainAmount_20_ = 0.85; β_GainAmount_30_ = 0.383; β_LossAmount_500_ = −0.3; β_LossAmount_750_ = 0.2 (the first column of Table 3). The second column of Table 3 shows the results of subtracting $\frac{\beta_{0}}{3}$ from the initial coefficients obtained by OLS regression. Following the “zero-centered diffs” procedure (Sawtooth Software, 2012), the means of new betas (from column 2) are then calculated for every attribute, with results as follows: Loss cards = 8.642; Gain amount = 10.257; Loss amount = 9.812. In column 3, the results of subtracting the means of each attribute from the transformed betas in column 2 are presented. Part-worth utilities are in column 4. These are calculated based on the ranges of betas in column 3 for each attribute: the range for Loss cards is 2.333; for Gain amount 0.850; and for Loss 0.500. The sum of the ranges is 3.683, so every beta in column 3 is multiplied by 81.448 (300/3.683) to render the sum of the three differences in the best and worst levels of each attribute equal to 300. This gives an average 100 point range in beta coefficients for each attribute.

**Table 3:** **Calculation of part-worth utilities**.

	**Beta (1)**	**Subtracting $\frac{\beta_{\text{0}}}{\text{3}}$ (2)**	**Subtracting means (3)**	**Part-worth utilities (4)**
Constant	29.537	–	–	–
Loss card (1)	–	9.846	1.204	98.039
Loss card (2)	–1.278	8.568	−0.074	−6.033
Loss card (3)	−2.333	7.512	−1.130	−92.006
Gain amount (10)	–	9.846	−0.411	−33.484
Gain amount (20)	0.850	10.696	0.439	35.747
Gain amount (30)	0.383	10.229	−0.028	−2.262
Loss amount (250)	–	9.846	0.033	2.715
Loss amount (500)	−0.300	9.546	−0.267	−21.719
Loss amount (750)	0.200	10.046	0.233	19.005

From the part-worth utilities we can find the utilities for any CCT round and can subsequently predict the participant's preferences between different CCT rounds. For example, the round characterized by 2 loss cards, gain amount equal to 20, and loss amount 750 has a utility of 48.715 (−6.033 + 35.747 + 19.005) and is less preferred than the round with 1 loss card, gain amount 10, and loss amount 250, which has a utility of 67.270 (98.039 + (−33.484) + 2.715).

Finally, the RIS for each attribute can be calculated. These are 63.35% for Loss cards, 23.07% for Gain amount, and 13.57% for Loss amount. RIS coefficients are obtained by rescaling ranges of part-worth utilities so that they sum to 100%. The ranges for part-worth utilities are as follows: 190.045 for Loss cards, 69.231 for Gain amount, and 40.724 for Loss amount, these summing to 300. Based on RIS values, we can conclude that for the participant whose decisions are presented in Table 1 the most important attribute of the CCT rounds is probability while gain amount is less important.

##### The cognitive reflection test (CRT)

The CRT uses three simple numeracy questions to assess individuals' ability to resist reporting the first response that comes to mind (generated by Type 1 processes/System 1) in favor of a reflective correct answer (generated by Type 2 processes/System 2). Each question is scored in a binary manner: correct = 1 and incorrect = 0, the total CRT score being the sum of these [a natural number between 0 and 3, representing a range from no correct answer (0) to three correct answers (3)]. Thus, higher scores imply a higher ability to resist implicit System 1 processes.

#### Participants

All participants gave their informed consent in accordance with the APA Ethical Principles of Psychologists and Code of Conduct, being fully debriefed at the end the study. Participants performed the study individually, in front of a computer separated from other PC stations by cubicles, providing privacy from other participants. Altogether *N* = 497 participants took part in Study 1 (73% females, aged *M* = 24.26; *SD* = 5.13), of which 380 were students of Cracow University of Economics and 117 were Kozminski University students. Students participated for course credits (with no monetary reimbursement) that depended on their CCT total score.

#### Procedure

The experiment used Inquisit (2012) by Millisecond Software (with a CCT script downloaded from the Inquisit Task Library). First, participants completed a prognostic strategy task (as described in Markiewicz et al., 2015)^3^ not related to this paper, and then the CCT in either its Hot or Cold version. Initially a long version of the CCT was used, with 63 rounds (27 × 2 + 9) in both the Hot and Cold conditions, and with three levels per parameter (1/2/3 loss cards, 10/20/30 gain amounts, 250/500/750 loss amounts). After the CCT, participants completed the CRT (Frederick, 2005) and a sociodemographic questionnaire.

Participants were randomly assigned to one of two conditions, taking either the Cold (*n* = 233, 74% females) or Hot (*n* = 264, 72% females) version of the CCT. A control analysis revealed that participants were balanced in terms of gender (χ^2^ = 0.335), although cold condition participants (aged *M* = 24.77; *SD* = 5.34) were slightly older [*t*_(471.263)_ = 2.060; *p* = 0.04) than hot condition participants (aged M = 23.81; *SD* = 4.90).

## Results

### Study 1 results

Results for the CCT risk taking propensity measure for the whole sample are presented in Table 4.

**Table 4:** **The general results of Study 1**.

**Study 1**	**CCT Cold, *n* = 233**		**CCT Hot, *n* = 264**		***t*-test**	***p***
	***M***	***SD***	***M***	***SD***		
Risk taking (M number turned over cards)	14.000	5.303	27.185	3.173	−32.985	0.000
Info use (*R*^2^)	0.425	0.202	0.338	0.235	4.448	0.000
Info use (ANOVA)	1.382	0.843	0.803	0.691	8.301	0.000
Loss amount decision weight (RIS)	0.304	0.161	0.253	0.143	3.748	0.000
Gain amount decision weight RIS	0.251	0.143	0.221	0.131	2.442	0.015
Probability decision weight (RIS)	0.446	0.186	0.527	0.211	−4.552	0.000
Loss amount INDEX (RIS ^*^R^2^)	0.120	0.084	0.068	0.048	8.339	0.000
Gain amount INDEX (RIS ^*^R^2^)	0.097	0.069	0.061	0.055	6.407	0.000
Probability INDEX (RIS ^*^R^2^)	0.208	0.153	0.209	0.207	−0.054	0.957

On average, respondents performing the Hot task disclosed more cards (*M* = 27.185; *SD* = 3.173) than those taking part in the Cold condition (*M* = 14.000; *SD* = 5.303). Those participating in the cold condition also displayed higher information use, regardless of measure employed (Table 4). The *R*^2^ measure, which we propose as a measure of information use, correlated highly with the ANOVA base ratio: *r*_(495)_ = 0.613; *p* < 0.001.

There was a difference in weights (RIS) associated with probability and payoffs at the general level (Table 4), indicating that Cold participants paid relatively more attention (as compared to Hot participants) to loss and gain amount importance and less to probability information.

However, taking into account the generally higher level of information use in the Cold condition, the above method of analyzing data may be misleading. It is likely that lower decision weights for probability in the Cold condition together with increased information use may constitute the same level of probability processing with an increased focus on the remaining decision characteristics. Detailed analysis supported this suspicion (Table 4): the probability index (RIS of probability ^*^R^2^) remained virtually the same for both the Hot and Cold conditions, while the loss amount index and the gain amount index were both highly increased in the Cold condition. Thus, the information use increase observed in the Cold condition was mostly caused by increased concentration on gains and losses in the Cold condition, while the focus on probabilities remained similar in the Cold and Hot conditions, supporting H1.

Hypothesis H2 suggested that information use would correlate with CRT scores. For the two conditions combined, this hypothesis was confirmed in respect of both the newly proposed *R*^2^ parameter [*r*_(495)_ = 0.182; *p* < 0.001] and the ANOVA parameter [*r*_(495)_ = 0.225; *p* < 0.001). The relationships were, however, specific to the Cold condition, and did not occur for the Hot condition (see Table 5 for details).

**Table 5:** **Correlations between CRT scores and information use (as measured by *R*^2^ and ANOVA ratios), separately for the Cold and Hot condition**.

**CRT correlation with…**	**Study 1**	
	**Cold, *n* = 233**	**Hot, *n* = 264**
Info use (*R*^2^)	0.303^**^	0.062
Info use (ANOVA)	0.349^**^	0.048
Loss amount decision weight (RIS)	−0.032	−0.153^*^
Gain amount decision weight (RIS)	−0.038	−0.003
Probability decision weight (RIS)	0.058	0.106
Loss amount INDEX (RIS ^*^R^2^)	0.226^**^	−0.049
Gain amount INDEX (RIS ^*^R^2^)	0.202^**^	0.034
Probability INDEX (RIS ^*^R^2^)	0.185^**^	0.073

Significance codes:

** *p < 0.01*,

* p < 0.05.

As shown in Table 6, decision weight (RIS) correlated highly with information use: the more a decision can be explained by risk parameters, the more decision makers care about probabilities, and less about pay-offs. This confirms the role of the loss probability parameter in both cold and hot processing.

**Table 6:** **Correlations between information use (as measured by *R*^2^ and ANOVA 5%) and task decision weights**.

**RIS**	**Info use *R*^2^**		**Info use ANOVA 5%**		**Info use *R*^2^**	**Info use ANOVA 5%**
**Study 1**	**Cold, *n* = 233**	**Hot, *n* = 264**	**Cold, *n* = 233**	**Hot, *n* = 264**	**All, *n* = 497**	**All, *n* = 497**
Loss amount decision weight (RIS)	−0.280^**^	−0.519^**^	−0.175^**^	−0.261^**^	−0.360^**^	−0.138^**^
Gain amount decision weight (RIS)	−0.331^**^	−0.444^**^	−0.012	−0.187^**^	−0.359^**^	−0.047
Probability decision weight (RIS)	0.497^**^	0.626^**^	0.160^*^	0.293^**^	0.514^**^	0.136^**^
**Study 2**	**Cold, *n* = 44**	**Hot, *n* = 198**	**Cold, *n* = 44**	**Hot, *n* = 198**	**All, *n* = 242**	**All, *n* = 242**
Loss amount decision weight (RIS)	−0.260	−0.082	0.069	−0.094	−0.128^*^	−0.068
Gain amount decision weight (RIS)	−0.497^**^	−0.293^**^	−0.299^*^	−0.257^**^	−0.342^**^	−0.275^**^
Probability decision weight (RIS)	0.625^**^	0.299^**^	0.161	0.282^**^	0.375^**^	0.271^**^

Significance codes:

** *p < 0.01*,

* p < 0.05.

### Study 1 interim discussion

Study 1 demonstrated that in affect-poor situations decision makers are more likely to focus on all risk parameters (and thus could rely on a compensatory process trading-off outcomes and probabilities), whereas in affect-rich choices people more often rely on a noncompensatory heuristic process (thus focusing on a single risk situation parameter—here, probability). This supports H1. One could however argue that in the current affect-rich situation (CCT Hot) people focused on probability because it was the only dynamically changing parameter. For example, having the information that there are 2 loss cards hidden among 32 cards, one knows that the probability of turning over a loss card is initially 2/32. After turning over the next card, the probability rises to 2/31, and after the third card to 2/30, etc. The other game parameters remain constant and do not change with each turned over card, it being a widely known phenomenon that people concentrate on change and not on states (Kahneman and Tversky, 1979). To control this we conducted Study 2 with delayed feedback in the CCT Hot condition.

### Study 2

Based on the above results verifying H1, we can state that probabilities are the most important risk parameter in the Hot task. Study 2 was conducted to provide better understanding of the role of probabilities while making risk decisions in the Hot task. We amended the CCT Hot procedure to freeze the probability parameter in a single round, making it constant for the whole of a round. Doing this allowed us to test whether the result of Study 1was determined by positive immediate feedback causing changes in the probability parameter each time a card was turned over.

#### Procedure

To address the issues raised in the previous section we used a delayed feedback procedure for the Hot condition^4^ (also developed by Figner, private communication), sometimes called the “CCT warm” condition (Huang et al., 2013). In the amended task a participant points to the cards to turn over (one after another), but needs to click a special button at the end of the round to turn over all cards pointed at (they are revealed in the order that they were pointed at). With this modification, participants need to make the decision before they see the sequence of turned over cards. Thus, the probability of loss does not change over a single round, and pointing to a card to turn over does not increase the probability of turning over a loss card on the next move.

Altogether, 242 Kozminiski University and Cracow University of Economics students (62% females, aged *M* = 26.87; *SD* = 4.90) took part in Study 2. The participants performed either the CCT Cold task (*n* = 44) or the CCT Hot delayed feedback task (*n* = 198)^5^. A control analysis revealed that participants were balanced in terms of age (*p* > 0.05), however females were over-represented in the Cold condition (86%) compared to the Hot delayed condition (57%). Since the Hot condition is usually more time consuming than the Cold condition, to save participants' time, and to make the duration of the two conditions more comparable, we developed a shorter CCT Hot delayed version. This had 32 rounds: 3 probability levels (1/2/3 loss cards) × 3 gain amounts (10/20/30) × 3 loss amounts (250/500/750) = 27 rounds, plus 5 rigged rounds distributed randomly. Thus, the shortening involved only the number of task rounds (similar to Huang et al., 2013; Holper and Murphy, 2014) but not the number of levels as in Figner and Weber (2011), Panno et al. (2012), Penolazzi et al. (2012), and Buelow (2015).

Because of the amendment, the results for the Study 2 CCT Hot condition cannot be directly compared to those of Study 1. Study 2 was conducted to verify the role of the probability parameter in the Hot task, which is specifically related to H1, thus participants did not complete the CRT (Frederick, 2005) which related only to H2. As in Study 1, students participated for course credits, which depended on their total CCT performance. The students participating in studies 1 and 2 were mutually exclusive.

#### Results

Table 7 below presents the results of Study 2. In the Hot delayed condition, participants demonstrated comparable information use levels to the Cold condition (as measured both by ANOVA and *R*^2^ based ratios), with no significant differences in the number of turned over cards.

**Table 7:** **The general results of Study 2**.

**Study 2**	**CCT Cold, *n* = 44**		**CCT Hot delayed, *n* = 198**		***t*-test**	***p***
	***M***	***SD***	***M***	***SD***		
Risk taking (M number turned cards)	15.076	6.519	14.823	6.448	0.235	0.815
Info Use (*R*^2^)	0.614	0.618	0.783	0.603	−1.676	0.095
Info use (ANOVA)	0.841	0.608	1.051	0.746	−1.740	0.083
Loss amount decision weight (RIS)	0.343	0.175	0.308	0.144	1.374	0.171
Gain amount decision weight (RIS)	0.297	0.139	0.251	0.129	2.105	0.036
Probability decision weight (RIS)	0.360	0.183	0.441	0.165	−2.853	0.005
Loss amount INDEX (RIS ^*^R^2^)	0.130	0.090	0.145	0.096	−0.970	0.333
Gain amount INDEX (RIS ^*^R^2^)	0.107	0.054	0.113	0.071	−0.480	0.632
Probability INDEX (RIS ^*^R^2^)	0.166	0.156	0.221	0.138	−2.322	0.021

The fact that the Hot delayed condition and the Cold version of the CCT do not differ significantly in terms of total information use shows that blocking positive feedback in the Hot version makes it similar to the Cold version. Although there were some differences in participants' treatment of certain risk attributes, Cold condition participants paid more attention (compared to Hot participants) to gain amount and less to probability information.

Detailed analysis of parameter contributions toward *R*^2^ (Table 7) showed that information use in the delayed Hot feedback condition grew mostly through a focus on probability (compared to the Cold condition) with a relatively similar focus on loss and gain amounts. Thus, even after desensitizing participants to permanent changes in probabilities (by replacing the Hot version with the Warm delayed feedback version), probability remained the most significant parameter. In the CCT Hot version participants concentrate on the distribution of every trial, i.e., the Bernoulli distribution, which involves the probability of success (a gain card) which is dependent on the number of cards already turned over (t), the number of loss cards (n) in the round: $\frac{32-t-n}{32-t}$, and the probability of failure (a loss card) which has a probability of $\frac{n}{32-t}$. Thus, in the CCT Hot task *t* changes with every turned over card, and the probabilities of success, and failure also change. In the CCT Warm version, probability is still important, but we suspect that participants consider a binomial distribution rather than a Bernoulli distribution. With every card selected, the probability of success and failure are the same, being equal to $\frac{32-n}{32}$ and $\frac{n}{32}$ respectively. A participant makes a decision about the total number of turned over cards, i.e., the number of independent Bernoulli trials in the binomial distribution, and is ultimately interested in the overall probability of turning over all success cards and no loss card.

In the more demanding Hot version of the CCT, the focus is on probabilities too. The Study 2 result cannot be attributed to changes in the risk parameters that are typical of the Hot task because in the Hot delayed condition positive feedback (which is the most important feature of the CCT Hot task) was disabled.

## General discussion

A set of two experiments yielded data supporting both hypotheses. The CRT (Frederick, 2005) is considered to be a tool measuring the cognitive ability to suppress an intuitive and spontaneous (Type 1 processes) wrong answer in favor of a reflective and deliberative (Type 2 processes) correct answer. We showed that CCT information use (measured using both ANOVA and OLS based indices) correlates with CRT score only in the Cold condition and *not* in the Hot condition. This means that the ability to use Type 2 processes is a good predictor of information usage in CCT Cold, but NOT in Hot, processing. When cognition is flooded with affect (in affective tasks) the aforementioned ability is not related to information processing. Thus, H2 was supported. Moreover, the result provides an argument that the CCT Cold task requires mostly Type 2 processes, while the CCT Hot task is dominated mostly by Type 1 processes.

We have also suggested a new method of information use parameter calculation. The original method (Figner et al., 2009) is based on ANOVA and the outcome parameter is measured on a scale ranging from 0 to 3 (0 = no parameters taken into account, 3 = all three parameters taken into account). In doing this, however, the researcher cannot say—at the individual level—which risky situation parameter (probability, gain amount, loss amount) influences the decision maker most, e.g., the researcher knows that both probability and degree of loss influence a decision, but they do not know which parameter is more important for the decision maker. As a methodological insight, we employed a conjoint analysis methodology (Orme, 2010) to propose a new way of measuring information use. Thus, analyzing individual choices with OLS regression (as opposed to the ANOVA method proposed by Figner et al., 2009) provides an individual respondent-specific *R*^2^coefficient reflecting the magnitude of information's influence on risk taking. Additionally, we have suggested further mathematical transformations of regression betas to obtain: (1) the equivalent of conjoint utilities (part-worths) for each information level and (2) the average relative importance score (referred to as decision weights, summing up to 100%) for each of the three task parameters–loss probability, gain amount, and loss amount. We can therefore compare the influence of each information type on decision making—and make comparisons between respondents. We used the new information use parameter to verify H1.

As demonstrated, in the Cold condition decision makers concentrate on each information type (gain, loss, probability), while in the Hot condition concentration on probabilities virtually monopolizes the decision making process, making loss, and gain amount almost unnoticeable. Making a decision based on one risk parameter in the Hot CCT task is simpler and more heuristic in nature than making one based on three parameters in the Cold CCT task. This supports H1.

The dominance of one parameter in the Hot task (heuristic, simplified decision making), as compared to more comprehensive information processing focusing on all available information, was an expected result. Similar dominance of one dimension of a problem over another can be found in studies of moral judgments where an individual faces a conflict between moral rules and the consequences of alternative actions. In these types of problem, people analyze the consequences more when affective arousal is low (indirect action), but when arousal is high they usually rely on their moral intuitions (direct action, direct contact: Paharia et al., 2009; Białek et al., 2014). Previous studies (Pachur et al., 2014; Suter et al., 2015) have demonstrated that in the context of outcomes laden with affective responses the traditional assumption of expectation maximization may not apply. While the current study is in line with this notion, it yielded opposite results regarding probability importance in hot vs. cold risk taking situations. While Pachur's study and other studies have shown a diminished role of probability information in the Hot task, our study ran contrary, demonstrating an increased role of probability in the Hot task. A couple of factors can explain this difference. It is worth noting that the previous studies (Rottenstreich and Hsee, 2001; Pachur et al., 2014; Suter et al., 2015) used different modality stimuli for affect-rich and affect-poor outcomes (e.g., medical vs. monetary, pain vs. monetary, etc.) to show that for affect-rich outcomes people refrain from focusing on the probability of outcomes. This result could, however, stem from both an affect load difference between conditions and domain differences (some domains are more naturally suited to concentrating on probabilities than others (Sawicki and Markiewicz, 2015). Thus, we believe that researching the effect of affect-rich and affect-poor outcomes within one domain (here: monetary) is critical. After doing this, we demonstrated that in a Hot task people act in a simplified way, concentrating on only one parameter, mostly probability. So, for affect-rich choices the process of weighing possible outcomes by their probabilities is biased.

Our results are in line with other CCT studies—e.g., Buelow (2015, p. 184) says “*It appears that participants in the CCT-cold condition paid greater attention to all three information use variables in making decisions than those in the CCT-hot condition, in which riskier performance was only associated with loss probability*.”

We believe that this result can be potentially explained in two other ways related to task specificity:

1. The Hot and Cold CCT tasks employed did not only differ in the affect load of their outcomes. The greater affect load of the CCT Hot outcomes, as compared to the Cold outcomes, was obtained by providing immediate feedback to respondents in the Hot task (Loewenstein et al., 2001). This makes the whole situation more dynamic (Wallsten et al., 2005; Weber and Johnson, 2008). Thus, the real probability of loss dynamically changes after each turned over card. On the other hand, many theories (the most prominent example being prospect theory: Kahneman and Tversky, 1979) predict that people concentrate not on states, but on changes relative to a reference point. Thus, decision makers could concentrate on probability since it is the only parameter changing dynamically within a single round. However, by postponing feedback in Study 2 we demonstrated that even when the dynamic nature of the task is excluded, a similar effect of increased probability concentration in the Hot task (relative to the Cold task) is obtained.
2. Tyszka and Zaleskiewicz (2006) hypothesized that people are less sensitive to probabilities when they are faced with a single-choice situation than when they are faced with a series of lotteries with different probabilities. Thus, treating each card click in the Hot task as a separate lottery could make the whole task similar to a “series of lotteries” type of task, sensitizing decision makers to probabilities, and making probability a more salient parameter. Although Tyszka and Zaleskiewicz's (2006) study did not manage to positively verify their hypothesis, they provided a valuable theoretical justification using the “evaluability hypothesis” (Hsee, 1996; Hsee and Leclerc, 1998). In a series of studies Hsee demonstrated that attributes perceived as difficult to evaluate may become evaluable through direct comparison, such attributes eventually becoming more important in the decision process. Quite simply, the possibility of direct comparison of options differing in some feature increases the importance of the feature relative to situations where options are presented in isolation. The evaluability hypothesis can be applied to the CCT Hot task, which makes the probability parameter more salient. As noted by Tyszka and Zaleskiewicz “The probability parameter can be difficult to evaluate, particularly by people who have little experience with probabilistic judgment (…). When an individual is confronted with the same scenario several times, and the only parameter that changes is probability, the individual has a possibility to compare the target probability level with other levels of probability and to attach more weight to it” (p. 1630).

The above interpretation of the observed increase in focus on probabilities in the dynamic, repeated CCT Hot task makes a practical contribution in the area of possible corrective actions. Even if we know that decision makers often neglect probabilities for affect-rich outcomes (Sunstein, 2002), the repeated choice method seems to be a perfect tool to resensitize decision makers to probabilities. The problem of ignoring probability is important–as expressed by Suter et al. (2015) “…  to the extent that people show strongly attenuated sensitivity to probability information (or even neglect it altogether) in decisions with affect rich outcomes, different decision aids may be required to help them make good choices. For instance, professionals who communicate risks, such as doctors or policy makers, may need to pay special attention to refocusing people's attention on the probabilities of (health) risks by illustrating those risks visually” (p. 19). Presently, we demonstrated that repeated choice is also an efficient way of focusing attention on probabilities. It is worth noting that the repeated choices in the CCT Hot task can be interpreted as an “experienced probability” format for presenting outcome frequencies (Weber, 2006; Tyszka and Sawicki, 2011). Studies show that, compared to using percentages or an iconographic format, such a format allows people to ascertain probabilistic information more easily, even where the situation involves affect. Tyszka and Sawicki's (2011) Study 2 “… showed that for the emotionally laden stimuli, the experience-based probability format resulted in higher sensitivity to probability variations than other formats of probabilistic information. These advantages of the experience-based probability format are interpreted in terms of two systems of information processing: the rational deliberative vs. the affective experiential and the principle of stimulus-response compatibility” (p. 1832).

We have made a methodological advance in employing a conjoint analysis methodology (Orme, 2010) as a new way of measuring information use in the CCT, demonstrating its further usefulness in judgment and decision making studies (Czupryna et al., 2014; Bialek et al., 2015). We believe that this can be potentially useful in future studies investigating decision weights for particular information types in the CCT. Previous methods of computing the information use parameter would not have allowed the present conclusions to be reached.

### Conflict of interest statement

The authors declare that the research was conducted in the absence of any commercial or financial relationships that could be construed as a potential conflict of interest.

## Footnotes

^1^There is also a version of the CCT where every parameter can take one of two levels (10 and 30 for gain amount, 1 and 3 for number of loss cards, 250 and 750 for loss amount), e.g., Figner and Weber (2011), Panno et al. (2012), Penolazzi et al. (2012), and Buelow (2015).

^2^Official webpage with demo version: http://www.columbiacardtask.org.

^3^This task was performed for the purposes of another study not related to the current paper. The task did not involve a manipulation.

^4^The script can be downloaded from the Inquisit Task library.

^5^The unequal group sizes were intentional given the analytical focus of the new CCT Hot delayed methodology. We employed random sampling with 1 cold participant for every 5 recruited for Study 2.
